# Trial Watch: Adoptive TCR-Engineered T-Cell Immunotherapy for Acute Myeloid Leukemia

**DOI:** 10.3390/cancers13184519

**Published:** 2021-09-08

**Authors:** Diana Campillo-Davo, Sébastien Anguille, Eva Lion

**Affiliations:** 1 Laboratory of Experimental Hematology, Vaccine & Infectious Disease Institute (VAXINFECTIO), Faculty of Medicine and Health Sciences, University of Antwerp, 2610 Wilrijk, Belgium; sebastien.anguille@uantwerpen.be; 2 Center for Cell Therapy & Regenerative Medicine, Antwerp University Hospital, 2650 Edegem, Belgium; 3 Division of Hematology, Antwerp University Hospital, 2650 Edegem, Belgium

**Keywords:** T-cell receptor, TCR engineering, adoptive T-cell therapy, acute myeloid leukemia

## Abstract

**Simple Summary:**

Acute myeloid leukemia (AML) is a type of blood cancer with an extremely grim prognosis. This is due to the fact that the majority of patients will relapse after frontline treatment. Overall survival of relapsed AML is very low, and treatment options are few. T lymphocytes harnessed with antitumor T-cell receptors (TCRs) can produce objective clinical responses in certain cancers, such as melanoma, but have not entered the main road for AML. In this review, we describe the current status of the field of TCR-T-cell therapies for AML.

**Abstract:**

Despite the advent of novel therapies, acute myeloid leukemia (AML) remains associated with a grim prognosis. This is exemplified by 5-year overall survival rates not exceeding 30%. Even with frontline high-intensity chemotherapy regimens and allogeneic hematopoietic stem cell transplantation, the majority of patients with AML will relapse. For these patients, treatment options are few, and novel therapies are urgently needed. Adoptive T-cell therapies represent an attractive therapeutic avenue due to the intrinsic ability of T lymphocytes to recognize tumor cells with high specificity and efficiency. In particular, T-cell therapies focused on introducing T-cell receptors (TCRs) against tumor antigens have achieved objective clinical responses in solid tumors such as synovial sarcoma and melanoma. However, contrary to chimeric antigen receptor (CAR)-T cells with groundbreaking results in B-cell malignancies, the use of TCR-T cells for hematological malignancies is still in its infancy. In this review, we provide an overview of the status and clinical advances in adoptive TCR-T-cell therapy for the treatment of AML.

## 1. Introduction

Acute myeloid leukemia (AML) is a type of blood cancer that carries a grim prognosis, despite considerable therapeutic advances in the last decade. Current treatment of newly diagnosed AML generally consists of intensive chemotherapy (IC) followed by allogeneic hematopoietic stem cell transplantation (allo-HSCT) in younger and fit patients, and low-intensity chemotherapy (e.g., hypomethylating agents) combined with—depending on the availability of the drug—the oral BCL-2 inhibitor venetoclax in older and/or less fit patients. Allo-HSCT, which is considered an immunotherapeutic strategy, since part of its mode of action involves the administration of T-lymphocytes that can recognize and eliminate the leukemic cells, is the gold standard post-remission treatment in AML. As discussed above, it is generally reserved for younger patients. Only a small population of elderly AML patients actually receives allo-HSCT [1]. Nevertheless, even with allo-HSCT, the majority of AML patients will relapse, explaining the poor 5-year overall survival rate of only 30% [2]. This explains why there is still a high unmet need to treat relapsed (or refractory) disease or to prevent relapse by strategies aimed at eradication of measurable residual disease (MRD; known before as minimal residual disease), which is the primary cause of relapse [3,4].

**Figure 1:**
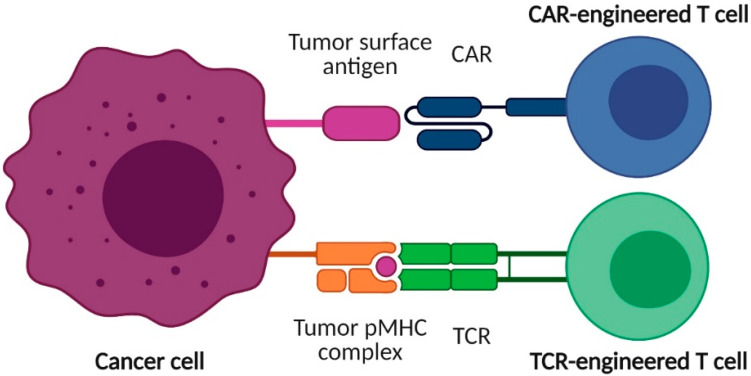
Chimeric antigen receptor (CAR) and T-cell receptor (TCR) engineered T cells. CAR-T cells usually target surface antigens, whereas TCR-T cells recognize internally processed proteins presented by molecules of the major histocompatibility complex (MHC) as peptide-MHC (pMHC) complexes. Each TCR is specific for a pMHC, allowing an infinite set of pMHC combinations that can be exploited for TCR-T-cell therapy. Created with BioRender.com (San Francisco, CA, USA).

It is within this context that immunotherapy comes to the fore. So far, most clinical trials with immunotherapies in AML have focused on monoclonal antibodies or checkpoint inhibitors. Cell-based immunotherapies for AML, except for allo-HSCT, are still experimental and have not yet surpassed the clinical trial stage [5]. Among the cell therapies that are currently being studied in AML are dendritic cells (DC) loaded with leukemia-associated antigens to stimulate anti-leukemia (T-cell) immunity; DC “vaccination” has shown promising results, especially as maintenance therapy in a low-disease burden setting [6]. Still in their infancy but promising are clinical trials that explore adoptive cell therapy (ACT) with leukemia-reactive T lymphocytes. AML cells are known to express a broad range of tumor antigens, including—but not limited to—overexpressed leukemia-associated antigens (LAAs), leukemia-specific antigens (LSAs) or neoantigens and cancer-testis antigens (reviewed by [7,8]). Target antigens are either molecules expressed on the surface (“extracellular”) or internally processed (“intracellular”) proteins that are present on the AML surface in the form of peptides bound to human leukocyte antigen (HLA)/major histocompatibility complex (MHC) molecules, which can be recognized by T cells via their T-cell receptor (TCR) (Figure 1). Surface (“extracellular”) antigens are targeted by chimeric antigen receptor (CAR) T-cell therapy. This form of therapy, which involves the adoptive transfer of lymphocytes genetically modified to express a CAR, has become an established treatment option in different hematological malignancies [9,10,11]. CAR-T-cell therapy has also been aimed at treating AML (reviewed by [12,13,14]). The first clinical report of CAR-T-cell therapy in AML dates back to 2013; in that study, a second-generation CAR against Lewis Y antigen showed limited clinical efficacy, but showed T-cell biological activity in terms of trafficking to the BM and in vivo persistence without hematopoietic toxicity [15]. Other target antigens in anti-AML CAR T-cell therapy include CD33, CD123, and C-type lectin-like molecule-1 (CLL-1) [16]. Unfortunately, most of the “extracellular” target antigens in AML are also expressed on normal hematopoietic cells, posing an important barrier to the applicability of CAR-T-cell therapies in AML.

Although intracellular antigens can also be targeted by CARs using antibodies recognizing peptide-MHC (pMHC) complexes [17], traditionally, this has been the role of TCRs. Thus, in TCR-T-cell therapies, and similar to CAR-T-cell therapies, lymphocytes for adoptive transfer are genetically engineered with tumor antigen-specific TCRs to redirect their specificity towards pMHC complexes (reviewed by [18] in solid tumors and by [19,20] in hematological malignancies). Conventional αβ T cells are the most frequent TCR-engineered cell type. More recently, other lymphocytes, such as γδ T cells, have garnered attention due to their excellent natural antitumor properties that can be exploited as cellular immunotherapy [21]. γδ T cells are a subset of unconventional T cells that express γδTCRs instead of αβTCRs and compose up to 10% of peripheral T cells [22]. Despite the low frequency in peripheral blood, these cells can be easily expanded ex vivo [23]. Given the numerous intracellular antigens identified in AML and the presence of leukemia-associated surface antigens in healthy cells [7,8], AML might be more suited for TCR-T-cell therapies. Moreover, compared to CARs, TCRs also require lower epitope densities to function [24]. Although there are certain drawbacks to the use of TCR-T cells, including their HLA restriction, which limits their broad applicability, TCR-based T-cell therapies have produced encouraging results, especially in patients with melanoma and certain other solid tumors [24]. In this review, we summarize the status and clinical advances in the use of TCR-engineered T cells for the treatment of AML.

## 2. Target Antigens in TCR-T-Cell Therapies for AML

Cancer immunotherapies using TCR-T cells require TCRs that recognize tumor antigens in a specific manner [25]. Currently, Wilms’ tumor 1 (WT1), preferentially expressed antigen in melanoma (PRAME), and minor histocompatibility antigens (MiHA) have been the only AML antigens targeted using TCR-T cells in a clinical setting.

### 2.1. WT1

WT1 is a zinc-finger translation factor that is overexpressed in AML and other hematological and solid malignancies [26]. It is mainly found in the cytoplasm of tumor cells [27,28], whereas in leukemic cells, it is more frequently found in the nuclei [27,29], although it can shuttle between the nucleus and cytoplasm [30]. In AML, WT1 overexpression occurs in 73–93% of patients at diagnosis (reviewed by [31]). Although other markers and techniques are preferred for MRD measurement (reviewed by [3,4]), *WT1* messenger RNA (mRNA) levels can be used as a marker for MRD and a prognostic factor for relapse in AML if no other markers are available [31]. Somatic *WT1* mutations occur in approximately 6–15% of AML patients at diagnosis (reviewed by [32]) and are more frequent at a younger age and in cytogenetically normal AML (CN-AML) patients [31,32], which account for 40–50% of AML patients [33,34]. *WT1* aberrations usually occur in exons 1, 7, and 9, primarily creating premature stop codons and frameshift mutations affecting the reading frame [32]. These mutations appear to confer a negative prognostic outcome by increasing the risk of relapse and death.

### 2.2. PRAME

PRAME is a repressor of the retinoic acid receptor [35]. Similar to WT1, PRAME is also overexpressed in different cancer types, including AML [36]. Approximately 30–87% of patients at diagnosis overexpress *PRAME* mRNA [36,37,38], and, as with WT1, it could be used as a surrogate marker of MRD in AML [37]. Combined detection of WT1 and PRAME has been suggested to be a sensitive molecular biomarker for monitoring MRD in AML [39]. CD8 T-cell responses against WT1 and PRAME are detected in healthy individuals and AML patients [40,41,42,43]. More importantly, multi-epitope WT1-specific and PRAME-specific T-cell reactivities have also been confirmed in clinical studies after vaccination with WT1-loaded DC [44] or PRAME peptide vaccination [45].

### 2.3. MiHA

MiHA antigens are polymorphic peptides presented by HLA molecules, resulting from the degradation of proteins from polymorphic genes with single-nucleotide polymorphisms, base-pair insertions or deletions, or copy number variations [46]. In cancer therapy, allogeneic T cells from an HLA-matched donor—administered in the setting of allo-HSCT or donor lymphocyte infusions—can recognize mismatched MiHA in the recipient patient [46]. When this recognition occurs against MiHA expressed by the leukemic cells, donor T cells induce graft-versus-leukemia (GVL) processes; however, if the polymorphic peptide is also expressed in normal cells, undesirable graft-versus-host disease (GVHD) can also occur [46]. In hematological malignancies, donor T cells from donor lymphocyte infusions targeting the ubiquitously expressed MiHA peptides can induce complete remissions by GVL, albeit frequently accompanied by concurrent GVHD [47]. In contrast, T cells recognizing hematopoiesis-restricted MiHA peptides, i.e., only expressed in cells of hematopoietic origin, such as HA-1 and HA-2, can lead to complete remissions in leukemia patients that relapsed after receiving allo-HSCT by inducing GVL without severe GVHD [47]. This nonameric peptide has two allelic variants codified on chromosome 9, HA-1H and HA-1R, which vary in one single amino acid [48]. Both variants are able to bind HLA-A*02:01 molecules; however, only HA-1H can effectively be expressed on the cell membrane by HLA-A*02:01 and induce T-cell responses in HA-1 R/R homozygous individuals [48,49]. HA-1H variant, which is present in 30% of the population [49], can also be presented by HLA-A*02:06 [50].

## 3. Characteristics and Results of Clinical Trials Using AML-Directed TCR-T Cells

Currently, there are 12 early phase clinical trials with TCR-T cells against relapsed/refractory (R/R) AML, focusing on investigating the feasibility and safety of the therapy (Table 1, Table 2, Table 3 and Table 4). So far, 38 AML patients have been treated out of 57 hematological cancer patients recruited, and approximately 250 patients are intended to be recruited in total (status of trials listed in Table 1). Most of these clinical trials genetically engineer conventional autologous T cells with αβ TCRs to target WT1, PRAME, and MiHA in the context of HLA-A*02:01 restriction, which is expressed in approximately 50% of the European population (characteristics of T-cell products are listed in Table 2).

Five studies have focused on targeting WT1, of which three have been recently completed. Two of these three completed clinical trials (clinicaltrials.gov identifier: NCT01621724, NCT02550535) used escalating doses of HLA-A*02:01-restricted WT1-specific TCR-T cells (patient characteristics and treatment regimens are listed in Table 3) [51,52,53]. In both cases, single doses of either 2 × 10^7^ TCR-T cells/kg or 1 × 10^8^ T cells/kg were administered per cohort, accompanied by an injection of interleukin (IL)-2. In NCT01621724, WT1-specific T cells persisted one year after infusion in four out of a total of seven patients with AML and chronic myeloid leukemia (CML) (outcomes of clinical trials using TCR-T cells for AML are listed in Table 4) [51]. Complete responses were also observed in four out of seven patients; however, it is unclear whether those with complete responses presented persistent WT1-specific T cells in peripheral blood. In NCT02550535, a total of six AML patients, three patients with myelodysplastic syndrome (MDS), and a patient with tyrosine kinase inhibitor-resistant CML were treated with a WT1_126-134-_specific TCR-T-cell product [52,53]. AML patients in complete morphological remission before treatment were administered a single dose of TCR-T cells accompanied with IL-2. TCR-T cells proliferated in vivo and were detectable 28 days following infusion in all patients. Median overall survival (OS) of AML patients following treatment with TCR-T cells was 12 months. It remains unclear whether patients with the highest median OS received the highest dose in the trial or not. In both studies, TCR-T-cell products were generally well tolerated. No adverse effects were observed, except a case of febrile neutropenia and cytokine release syndrome that was successfully treated.

While the HLA-A*02:01 allele is one the most frequent HLA class I types in European and North American populations, HLA-A*24:02 is the most common HLA-A allele in Japan. Therefore, the third completed phase I dose-escalation trial conducted in Japanese individuals focused on administering two rounds of an HLA-A*24:02-restricted WT1_235-243_ peptide-specific TCR to HLA-A*24:02-positive patients, followed by WT1 peptide vaccination (umin.ac.jp Identifier: UMIN000011519; [54]). In these three completed studies, TCR-T-cell treatment was well tolerated, with no dose-limiting toxicities related to treatment; however, one instance of cytokine release syndrome was reported, possibly related to treatment (Table 4). TCR-T-cell treatment was well tolerated, with seven out of eight patients showing no or only grade 1 adverse events. Observed adverse events were mostly related to peptide vaccination at the site of injection. WT1-specific TCR-T cells were successfully detected in the peripheral blood of eight treated patients. More importantly, TCR-T cells were still detectable 8 weeks post-administration in five of the eight treated patients. The number of circulating TCR-T cells positively correlated with the number of cells administered, especially during the first 14 days after treatment. WT1-specific TCR-T cells from one patient responded to WT1_235-243_ peptide, indicating that TCR-T-cell antitumor activity was still intact despite decreasing circulating T-cell numbers. A transient decrease in peripheral blood or BM blasts was observed in three cases after treatment, whereas the disease progressed in four patients. Median OS was 15.9 months, with four out of five patients with persistent T cells surviving longer than 12 months, compared to only one out of three patients without detectable engineered T cells surviving beyond 12 months. Further studies including more patients would be needed to completely ascertain the hematologic benefit of these WT1-specific TCR-T cells, especially since T cells were engineered with a TCR with a physiological affinity. It is also important to note that patients with decreasing blast numbers in BM after treatment were those with a higher percentage of WT1-specific TCR-T cells in the T-cell product. Therefore, achieving sufficient TCR-engineered T cells in the medicinal product is crucial in this type of therapy. Two other studies focusing on WT1 are still ongoing in the United States of America (USA; Table 1; NCT01640301 and NCT02770820) [55,56]. Both are centered on high-risk AML and use the same HLA-A2-restricted T-cell product called WT1-T_TCR-C4_ (Table 2), followed by subcutaneous injection of IL-2 (Table 3). Preliminary results from 12 treated patients (NCT01640301) indicate that treatment was generally well tolerated (Table 4) [55]. GVHD was observed in some patients, including a case of grade 3 acute GVHD; however, since the onset of GVHD events occurred at a median of 123 days after infusion, GVHD was likely not caused by the T-cell product. TCR-T cells persisted in 75% of patients until day 28 post-administration and were detected after 12 months in 33% of patients. More importantly, clinical efficacy following WT1-specific TCR-T-cell treatment was demonstrated by a relapse-free survival (RFS) of 100% at a median of 44 months, which was significantly higher than a comparable group of high-risk AML patients that did not receive TCR-T-cell therapy [55]. Results from trial number NCT02770820 regarding the persistence of T cells and disease response are currently not available. Preliminary data on adverse events indicate that TCR-T cells were well tolerated, with no severe adverse effects (Table 4) [56]. Only four out of seven patients completed treatment. One patient died during treatment; however, due to the absence of data on the cause of death, it is difficult to determine whether it was related to treatment or not.

With regard to PRAME-specific TCR-T cells, two different trials are ongoing for relapsed AML in the context of HLA-A2 restriction (Table 1). Both NCT02743611 and EudraCT-2017-000440-18 trials are analyzing increasing doses of autologous TCR-T-cell products (BPX-701 and MDG1011, respectively) up to 5 × 10^6^ T cells/kg (Table 2 and Table 3) [57]. In addition, in a third clinical study (EudraCT-2018-000717-20), patients enrolled in trial EudraCT-2017-000440-18 will be followed up for up to 14 years. Results from these clinical trials are pending and will provide the field with information about the future of PRAME-specific TCR-T-cell therapies in AML.

**Figure 2:**
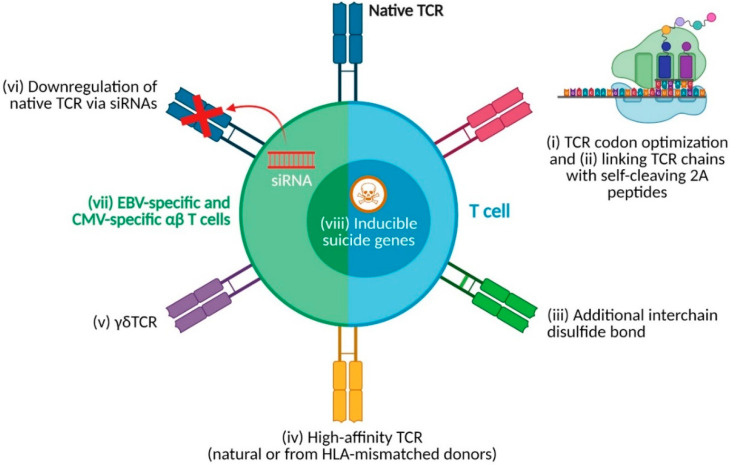
TCR-directed optimization strategies that have been clinically tested in AML. Different strategies to enhance TCR-T-cell therapies have been used in clinical trials for the treatment of AML. These include (i) codon optimization of transgenic TCR sequences to improve protein translation; (ii) linking transgenic TCR alpha and beta chains via self-cleaving 2A peptides for equimolar expression of TCR chains; (iii) addition of cysteine residues that bind covalently to provide a second interchain disulfide bond and improve pairing of transgenic TCR chains; (iv) usage of high-affinity TCRs, either derived from normal TCR repertoires after screening of multiple TCR candidates or from HLA-mismatched donors to generate allo-restricted TCRs, to enhance antigen recognition; (v) TCR-engineering with γδTCRs derived from γδ T cell clones, or (vi) downregulation of native TCR chains via introduction of small interfering “silencing” RNAs (siRNAs) targeting native TCR transcripts to prevent TCR mispairing; (vii) usage of autologous donor-derived Epstein–Barr virus (EBV) and cytomegalovirus (CMV)-specific T cells to prevent EBV and CMV infections and graft-versus-host disease in TCR-T-cell treated patients; and (viii) introduction of inducible suicide genes that would allow the elimination of TCR-engineered T cells in case of treatment-related toxicities. Created with BioRender.com.

Two studies carried out in the Netherlands and one in the USA have targeted MiHA HA-1H peptide following allo-HSCT (Table 1). In the completed study (EudraCT-2010-024625-20), donor-derived Epstein-Barr virus (EBV) and/or cytomegalovirus (CMV)-specific T cells were engineered with an HLA-A*02:01-restricted MiHA HA-1H peptide-specific TCR [49,58], which contained an additional disulfide bond to improve TCR pairing and stability, in which the sequence was codon optimized [59]. Donor-derived EBV- or CMV-specific T cells can be safely used for adoptive transfer because (i) the reactivity of these T cells is known, (ii) these T cells help to prevent EBV and CMV infections, and (iii) they do not induce GVHD (Figure 2) [55,58,60]. A drawback of this strategy is that patients may not be seropositive for EBV and CMV. Even if they are, sufficient T cells for TCR-T-cell development may not be available when using autologous EBV- or CMV-specific T cells. As illustrated in EudraCT-2010-024625-20, in nine recruited patients, only two were EBV and CMV seropositive [49]. Moreover, although seven donors were EBV seropositive, HA-1H TCR-transduced CMV or EBV-specific T cells could be produced in five individuals [49]. Four out of five treated patients received two administrations of engineered T cells. TCR-transduced donor-derived EBV-specific T cells were well tolerated, with no toxicities related to treatment nor GVHD [49]. A study conducted in the Netherlands is currently investigating this strategy (trialregister.nl identifier: NTR6541). The T-cell product called TEG001 is based on retroviral transduction of a high-affinity Vγ9Vδ2 TCR derived from the natural repertoire of a healthy individual [61,62]. Although there are no clinical results available, a preclinical in vivo evaluation of TEG001 demonstrated that TEG001 eradicated primary AML blasts [63]. More importantly, after administration, TEG001 persisted up to 50 days in mice but did not target human cord blood-derived healthy hematopoietic cells. 

**Table 1:** Clinical trials using TCR-T cells for AML.

Clinical Trial Identifier	Location	Status (First Posted)	Phase	AML Status	Prior Treatment	AML Patients Recruited or Treated (Intended)	Ref.
**WT1-Specific**							
NCT01621724 EudraCT-2006-004950-25	UK	Completed (2012, completed in 2018)	I/II	AML	n.d.	7 treated (18)	[51]
NCT02550535 EudraCT-2014-003111-10	Belgium Germany UK	Completed (2015, completed in 2018)	I/II	Relapsed/ stable AML	HAT	10 pt. treated: 6 AML, 3 MDS and 1 TKI- resistant CML (25-30)	[52,53]
UMIN000011519	Japan	Completed (2013, completed in 2018)	I	R/R AML	n.d.	12 recruited, 8 treated	[54]
NCT01640301	USA	Active, not recruiting (2012)	I/II	High-risk/ relapsed AML	allo-HSCT	12 treated (45)	[55]
NCT02770820	USA	Active, not recruiting (2016)	I/II	High-risk non-M3 AML	Consolidation chemotherapy	7 treated (9)	[56]
**PRAME-Specific**							
NCT02743611	USA	Active, not recruiting (2016)	I/II	Relapsed AML	n.d.	(28)	[57]
NCT03503968 EudraCT-2017-000440-18	Germany	Recruiting (2018)	I/II	R/R AML	HAT and/or allo-HSCT	(92)	n.d.
EudraCT-2018-000717-20	Germany	Ongoing (2019)	Long-term follow-up of phase I	R/R AML	HAT and/or allo-HSCT	(52)	n.d.
**MiHA HA-1H-Specific**							
EudraCT-2010-024625-20 NTR3454/NL3307	Netherlands	Completed (2012, prematurely ended in 2018)	I	High-risk AML	allo-HSCT	9 recruited, 5 treated (20)	[49,58]
NCT04464889 EudraCT-2019-002346-20	Netherlands	Active, not recruiting (2020)	I	R/R AML	allo-HSCT	(29)	n.d.
NCT03326921	USA	Recruiting (2017)	I	Recurrent AML	allo-HSCT	(24)	n.d.
**Other**							
NTR6541/NL6357	Netherlands	Recruiting (2017)	I	R/R AML	n.d.	(18)	[61,62,63]

Abbreviations: allo-HSCT, allogeneic hematopoietic stem cell transplantation; AML, acute myeloid leukemia; CML, chronic myeloid leukemia; HAT, hypomethylating agent therapy; MDS, myelodysplastic syndrome; MiHA, minor histocompatibility antigen; n.d., no data; PRAME, preferentially expressed antigen in melanoma; R/R, relapsed/refractory; TKI, tyrosine kinase inhibitors; UK, United Kingdom; USA, United States of America; WT1, Wilms’ tumor 1. Last search on 25 July 2021.

**Table 2:** Characteristics of T-cell products.

Clinical Trial Identifier	Name of T-Cell Product	TCR		T-Cell Population	Ref.
		Restriction	High-Affinity/Avidity		
**WT1-Specific**					
NCT01621724 EudraCT-2006-004950-25	WT1 TCR-001	HLA-A2	n.d.	Autologous T cells	[51]
NCT02550535 EudraCT-2014-003111-10	n.d.	HLA-A2	n.d. (allo-restricted TCR)	Autologous T cells	[52,53]
UMIN000011519	n.d.	HLA-A24	No	Autologous T cells	[54]
NCT01640301	WT1-T_TCR-C4_	HLA-A2	Yes (from healthy individual)	Donor-derived EBV-specific CD8 T cells	[55]
NCT02770820	WT1-T_TCR-C4_	HLA-A2	Yes (from healthy individual)	Autologous central memory/naïve CD8 T cells EBV-specific T cells	[56]
**PRAME-specific**					
NCT02743611	BPX-701	HLA-A2	Yes (allo-restricted donor)	Autologous T cells	[57]
NCT03503968 EudraCT-2017-000440-18	MDG1011	HLA-A2	n.d.	Autologous T cells	n.d.
EudraCT-2018-000717-20	MDG1011	HLA-A2	n.d.	Autologous T cells	n.d.
**MiHA HA-1H-specific**					
EudraCT-2010-024625-20 NTR3454/NL3307	n.d.	HLA-A2	n.d.	Autologous donor-derived CMV- and/or EBV-specific T cells	[49,58]
NCT04464889 EudraCT-2019-002346-20	MDG1021	HLA-A2	n.d.	Autologous T cells	n.d.
NCT03326921	n.d.	HLA-A2	n.d.	CD4 and CD8 memory donor T cells	n.d.
**Other**					
NTR6541/NL6357	TEG001	n.a. (Vγ9Vδ2 TCR)	Yes	Autologous T cells	[61,62,63]

Abbreviations: CMV, cytomegalovirus; EBV, Epstein–Barr virus; HA-1H, HLA-A*02:01-restricted minor histocompatibility antigen 1 peptide variant H; HLA, human leukocyte antigen; HLA-A2, HLA-A*02:01; HLA-A24, HLA-A*24:02; MiHA, minor histocompatibility antigen; n.a., not applicable; n.d., no data; PRAME, preferentially expressed antigen in melanoma; TCR, T-cell receptor; WT1, Wilms’ tumor 1. Last search on 25 July 2021.

**Table 3:** Patient characteristics and treatment regimens.

Clinical Trial Identifier	Age of Patients	No. Patients per Arm or Cohort	Dosage per Cohort	Additional Treatments	Ref.
**WT1-Specific**					
NCT01621724 EudraCT-2006-004950-25	1 pt. 18-64 years 6 pt. ≥ 65 years	Cohort 1: 3 pt. Cohort 2: 4 pt.	Cohort 1: ≤2 × 10^7^ T cells/kg Cohort 2: ≤1 × 10^8^ T cells/kg	Standard conditioning; 10^6^ units/m^2^ IL-2	[51]
NCT02550535 EudraCT-2014-003111-10	n.d.	Cohort 1: 7 pt. Cohort 2: 3 pt. (6 AML, 3 MDS and 1 TKI- resistant CML in total)	Cohort 1: ≤2 × 10^7^ T cells/kg Cohort 2: ≤1 × 10^8^ T cells/kg	Subcutaneous low-dose injections of IL-2 (1 × 10^6^ units/m^2^)	[52,53]
UMIN000011519	1 pt. 18-64 years 7 pt. ≥ 65 years	Cohort 1: 3 pt. (1 AML and 2 MDS) Cohort 2: 3 pt. (MDS) (+2 pt. extracohort; 1 AML and 1 MDS) Cohort 3: 0 pt.	Cohort 1: two doses of 2 × 10^8^ cells Cohort 2: two doses of 1 × 10^9^ cells Cohort 3: two doses of 5 × 10^9^ cells Cells administered at day 0 and 28	Subcutaneous injection of 300 μg mutated WT1_235-243_ peptide at day 30 and 44	[54]
NCT01640301	8 pt 18-64 years 4 pt. ≥ 65 years	Treatment arm: 12 pt. Prophylactic arm: 12 pt.	12/12 pt.; one dose of 10^10^ T cells/m^2^ 7/12 pt.; second dose of 10^10^ T cells/m^2^ (administered if frequency of TCR-T cells was <3% of total peripheral CD8+ T cells)	Subcutaneous low-dose injection of IL-2	[55]
NCT02770820	4 pt. 18-64 years 3 pt. ≥ 65 years	Cohort 1: 7 pt. (4/7 pt. completed treatment)	Cohort 1: Two doses (day 0 and day > 21)	Subcutaneous injection of IL-2	[56]
**PRAME-Specific**					
NCT02743611	n.d.	n.d.	Escalating doses from 1.25 × 10^6^ T cells/kg up to 5 × 10^6^ T cells/kg to be explored	Rimiducid (in response to treatment-related toxicity)	[57]
NCT03503968 EudraCT-2017-000440-18	n.d.	n.d.	Cohort 1: target dose of 1 × 10^5^ T cells/kg Cohort 2: target dose of 1 × 10^6^ T cells/kg Cohort 3: target dose of 5 × 10^6^ T cells/kg Optional cohort 4: up to 1 × 10^7^ T cells/kg	n.d.	n.d.
EudraCT-2018-000717-20	n.d.	n.d.	Patients that were treated with MDG1011 TCR-T-cell product in EudraCT-2017-000440-18 trial	n.d.	n.d.
**MiHA HA-1H-Specific**					
EudraCT-2010-024625-20 NTR3454/NL3307	4 pt. 18-64 years 1 pt. ≥ 65 years	Cohort 1: 5 pt. (4 AML and 1 B-LBL)	Cohort 1: two doses of ≥3 × 10^6^ T cells (day 8 and 14 after allo-HSCT)	n.d.	[49,58]
NCT04464889 EudraCT-2019-002346-20	n.d.	n.d.	Cohort 1: target dose of 0.3 × 10^6^ T cells/kg Cohort 2: target dose of 1 × 10^6^ T cells/kg Cohort 3: target dose of 3 × 10^6^ T cells/kg	n.d.	n.d.
NCT03326921	n.d.	n.d.	n.d.	n.d.	n.d.
**Other**					
NTR6541/NL6357	n.d.	n.d.	n.d.	n.d.	[61,62,63]

Abbreviations: allo-HSCT, allogeneic hematopoietic stem cell transplantation; AML, acute myeloid leukemia; B-LBL, B-cell lymphoblastic leukemia; CML, chronic myeloid leukemia; HA-1H, HLA-A*02:01-restricted minor histocompatibility antigen 1 peptide variant H; IL-2, interleukin 2; MDS, myelodysplastic syndrome; MiHA, minor histocompatibility antigen; n.d., no data; PRAME, preferentially expressed antigen in melanoma; TKI, tyrosine kinase inhibitors; WT1, Wilms’ tumor 1. Last search on 25 July 2021.

**Table 4:** Outcomes of clinical trials using TCR-T cells for AML.

Clinical Trial Identifier	Treatment-Related Toxicities (Grade 1–2)	Treatment-Related Serious Adverse Events (Grade 3–4)	Persistence of T Cells	Disease Response	Ref.
**WT1-Specific**					
NCT01621724 EudraCT-2006-004950-25	No dose-limiting toxicity	Cohort 1: febrile neutropenia (1/3 pt.)	Cohort 1: 2/3 pt. at day 365 Cohort 2: 2/4 pt. at day 365	Cohort 1: CR (1/3 pt.); no response (2/3 pt.) Cohort 2: CR (3/4 pt.); no response (1/4 pt.)	[51]
NCT02550535 EudraCT-2014-003111-10	No dose-limiting toxicity	Possibly treatment-related cytokine release syndrome (1/10 pt.)	10/10 pt. at day 28 7/10 pt. at day 29-365	6 AML pt.: median survival of 12 months	[52,53]
UMIN000011519	No dose-limiting toxicity Facial edema, dermatitis, fever, phlebitis, arrhythmia, stomatitis (1/8 pt.) Skin reaction at peptide injection site (7/8 pt.)	None	Cohort 1: 2/3 pt. at day 58 Cohort 2: 3/5 pt. at day 58	Decrease of abnormal erythroblasts in PB (1/8 pt.); Decrease of blasts in BM (2/8 pt.); Stable disease (1/8 pt.); Progressive disease (4/8 pt.)	[54]
NCT01640301	None disclosed	Cytokine release syndrome (2/12 pt.) Lymphopenia (12/12 pt.) Trombocythopenia (2/12 pt.) Neutropenia (2/12 pt.) Anemia (7/12 pt.)	9/12 pt. at day 28 4/12 pt. at day >365	No evidence of disease (AML recurrence) at median follow-up of 44 months (12/12 pt.)	[55]
NCT02770820	Not disclosed if treatment related: Fatigue, alanine aminotransferase increased, hyperglycemia (1/6 pt.); Anemia, thrombocytopenia (2/6 pt.); Neutropenia, leukopenia (3/6 pt.); Hypertension (4/6 pt.); Lymphopenia (5/7 pt.)	Not disclosed if treatment related: Death (1/6 pt.)	n.d.	n.d.	[56]
**PRAME-Specific**					
NCT02743611	No results available yet				[57]
NCT03503968 EudraCT-2017-000440-18	No results available yet				n.d.
EudraCT-2018-000717-20	No results available yet				n.d.
**MiHA HA-1H-Specific**					
EudraCT-2010-024625-20 NTR3454/NL3307	None	None	3/5 pt. at day 14 after second infusion	Relapsed AML prior to infusion leading to death (1/5 pt.; 1/4 AML pt.); Infections during follow-up leading to death (2/5 pt.; ¼ AML pt.); No AML relapse and alive (2/4 pt.)	[49,58]
NCT04464889 EudraCT-2019-002346-20	No results available yet				n.d.
NCT03326921	No results available yet				n.d.
**Other**					
NTR6541/NL6357	No results available yet				[61,62,63]

Abbreviations: AML, acute myeloid leukemia; BM, bone marrow; CR, complete response; HA-1H, HLA-A*02:01-restricted minor histocompatibility antigen 1 peptide variant H; MiHA, minor histocompatibility antigen; n.d., no data; PB, peripheral blood; PRAME, preferentially expressed antigen in melanoma; TCR, T-cell receptor; WT1, Wilms’ tumor 1. Last search on 25 July 2021.

## 4. Strategies for Enhancing TCR-T-Cell Products

Some of the abovementioned clinical trials have used different strategies to optimize antigen recognition, TCR expression, and mechanisms to address potential safety concerns (Figure 2). Autologous T cells have been transduced with a codon-optimized WT1_126-134-_specific TCR construct (NCT02550535) [52,53]. Codon optimization is a technique in which synonymous codons replace codons in coding sequences to improve protein translation rates and enhance protein expression. However, growing evidence indicates that, despite unaltering coding sequences, codon optimization may alter how proteins fold, thus impacting post-transcriptional modifications and protein functionality (reviewed by [64]). In the same study, TCR alpha and beta sequences were linked via a self-cleaving 2A peptide derived from porcine teschovirus-1 [52,53]. Self-cleaving peptides, such as those from the foot-and-mouth disease virus or the abovementioned porcine teschovirus-1, allow the expression of multiple proteins using the same open reading frame [65]. On the one hand, this facilitates cell engineering, as only one vector has to be produced instead of one per sequence. On the other hand, it ensures stoichiometric independent expression of each protein. Nonetheless, careful design of sequences within the vector, including the selection of 2A peptides, the inclusion of additional spacers or furin sequences and the position of the sequences of genes of interest within the open reading frame, is key for correct protein production and function [66,67]. In the case of TCR expression, it has been observed that placing TCR alpha sequences downstream of 2A peptide sequences is preferred [68]. Transgenic TCR expression was further improved in NCT02550535 by an additional disulfide bond between TCR alpha and beta chains [52,53]. This extra bond located within the extracellular domain of the TCR constant regions induces correct pairing of transgenic TCR chains and, therefore, avoids TCR mispairing between native and transgenic TCRs while retaining transgenic TCR functionality [69]. These techniques have also been exploited in other clinical trials (EudraCT-2010-024625-20 [49,59]; UMIN000011519 [54,70]; NCT02743611 [57]).

TCR affinity plays an essential role in TCR-T-cell avidity, which in turn is critical for the efficacy and clinical benefit of TCR-T-cell products [25]. Compared to affinity-matured TCRs, which can lead to supraphysiological affinities and fatal cross-reactivities [71], natural high-affinity TCRs recognize self-antigens within physiological conditions. However, T-cell clones of high affinity against self-TAAs, such as WT1 and PRAME, are usually eliminated after negative selection in the thymus, reducing the number of tumor-reactive TCRs that could be used in TCR-T-cell therapies. Those that remain, normally of low or intermediate affinity, usually promote inferior T-cell responses to physiological epitope densities, which would partly explain tumor immune escape [25]. Thus, high-affinity TCRs with better pMHC binding capabilities commonly generate better T-cell responses [25]. Techniques to enhance TCR affinity, e.g., artificial affinity maturation, have been developed to improve pMHC binding. TCR-T-cell trials for AML have capitalized on high-affinity HLA-A*02:01-restricted TCRs directed against WT1 (NCT01640301 and NCT02770820) [55,56] and PRAME (NCT02743611) [57] (Figure 2). High-affinity WT1-specific TCRs were obtained from an HLA-A*02:01-positive individual (NCT01640301 and NCT02770820) [55,56]. Isolation of high-affinity TCRs from (healthy) donor repertoires is a laborious process, and artificial maturation of TCR affinity can lead to deleterious cross-reactivities. Therefore, an alternative is to isolate high-affinity allo-restricted TCRs from HLA-mismatched donors (Figure 2) [72]. In the context of AML therapy, allo-restricted TCRs have been used to recognize HLA-A*02:01-restricted WT1_126-134_ peptide (NCT02550535) [52,53] and HLA-A*02:01-restricted PRAME peptides (NCT02743611) [57]. In another study, instead of a high-affinity αβTCR, a high-affinity antigen-specific γδTCR was used (NTR6541) [61,62,63]. As mentioned previously, most studies focus on engineering conventional αβ T cells with αβTCRs. However, unconventional γδTCRs derived from γδ T cells are another source of tumor-specific TCRs (Figure 2). These γδTCRs are not HLA restricted and can be safely introduced in αβ T cells instead of classical αβTCRs, since, as opposed to αβTCR gene transfer in αβ T cells, mispairing between transgenic γδTCR chains and native αβTCR chains is unlikely due to preferential pairing [73,74]. With this approach, safety issues associated with mispaired αβ TCR combinations formed from transgenic and native TCRs in TCR-engineered αβ T cells are circumvented. Alternatively, native TCR expression can be eliminated using small interfering RNAs (siRNAs) against native TCR sequences (UMIN000011519; Figure 2) [54]. Short hairpin RNAs (shRNAs) were included in the vector containing the HLA-A*24:02-restricted WT1_235-243_-specific TCR construct to silence the expression of native TCRs and prevent TCR mispairing between native and transgenic TCR chains [54]. Transgenic TCR downregulation was prevented by codon optimization of the TCR sequence. Currently, other techniques such as CRISPR-Cas9 are gaining momentum in TCR-T-cell therapies to completely disrupt native TCR expression or even replace native TCR sequences with transgenic TCR sequences [75,76,77]. Recently, results from a phase I trial using CRISPR-Cas9-mediated disruption of native TCR and programmed cell death 1 (PD-1) sequences in combination with transduction of a TCR targeting cancer-testis antigen New York esophageal squamous cell carcinoma 1 (NY-ESO-1) have shown that this technique is feasible and safe (NCT03399448) [78]. However, this form of native TCR disruption has yet to be investigated for AML.

Other clinical studies benefit from donor-derived virus-specific T cells for TCR transduction, such as EBV and CMV-specific T cells (Figure 2, NCT01640301, NCT02770820, and EudraCT-2010-024625-20) [49,55,56,58]. These T-cell subsets can be commonly isolated, reduce the possibility of graft-versus-host disease (GVHD) by an endogenous TCR, and are naturally enriched for central memory T cells with enhanced in vivo persistence [55]. However, to circumvent the issue of low numbers of CMV seropositive patients, only EBV-specific T cells were used in NCT01640301 and NCT02770820 trials [55,56]. In these two studies, EBV-specific T cells were transduced with a high-affinity WT1-specific TCR derived from HLA-A*02:01-positive healthy donor repertoires [55]. In all current TCR-T clinical trials for AML, T cells are genetically engineered via viral transduction. This technique enables the stable expression of introduced TCRs, which raises concerns about potential insertional mutagenesis and persistent adverse effects caused by the stably engineered TCR-T cells. Results from a recent report in which investigators characterized the genomic integration profile of TEG001 following retroviral transduction showed that this approach does not induce malignant transformation of engineered T cells [79]. However, to address potential safety issues, such as insertional mutagenesis and off-target and on-target off-tumor reactivities, safety mechanisms that can be induced in case of severe toxicities have been introduced in some TCR-T-cell clinical trials (NCT02743611, NCT03326921) [57]. These mechanisms are based on engineering T cells with suicide genes, such as inducible human caspase-9 (iC9; Figure 2). This protein is a key initiator of apoptosis and is activated after dimerization. Activation of iC9 can be induced following administration of rimiducid, a chemical compound that induces iC9 dimerization, thus inducing apoptosis in TCR-T cells. This system is not exclusive to TCR-T-cell therapies and can also be used in cases of haploidentical HSCT with alloreplete haploidentical T cells for AML treatment [80]. In this setting, administered haploidentical T cells promote immune reconstitution in patients, while the iC9 system can eliminate the administered T cells in the case of GVHD [80]. Alternatively, the aforementioned CRISPR-Cas9 and other approaches, such as those based on RNA or transposon/transposase systems, which are not per se viral vector-based engineering systems, may be used to replace viral transduction. However, CRISPR-Cas9 and transposon/transposase systems also entail genome editing, similar to viral transduction. In contrast, RNA-based systems, in which transgenic *TCR*-encoding mRNA is transfected into T cells alone or in combination with siRNA-mediated downregulation of native TCR, represent a transient self-limiting approach with a potentially better safety profile [81,82,83]. Nonetheless, despite extensive preclinical data, these techniques are still emerging in the clinical setting.

## 5. Future Directions in TCR-T-Cell Therapy for AML

Preliminary results indicate that TCR-T therapies for AML are safe and TCR-T cells can persist in most patients. Promising clinical data suggest that this form of therapy may also be efficacious in preventing relapse in AML patients. However, completed and ongoing trials have faced some limitations (Table 5). In two instances, investigators were not able to recruit a sufficient number of patients, whereas, in two other cases, planned TCR-T-cell numbers for administration were not achieved. Moreover, in the clinical trial EudraCT-2010-024625-20, the low efficacy of the HA-1H-specific T-cell product observed in treated patients led to the early termination of the study. Therefore, results from ongoing trials from which no results are available will provide more information about the benefit of TCR-T-cell therapy in this context.

**Table 5:** Limitations of clinical trials using TCR-T cells for AML.

Clinical Trial Identifier	Description of Limitations
**WT1-Specific**	
NCT01621724 EudraCT-2006-004950-25	Enrolment into the study was terminated early due to difficulties in the recruitment of patients
NCT02550535 EudraCT-2014-003111-10	Enrolment into the study was terminated early in Germany due to difficulties in the recruitment of patients
UMIN000011519	T-cell numbers for Arm 2 were not feasible for all patients; T-cell products were not feasible for Arm 3
NCT01640301	None disclosed
NCT02770820	None disclosed
**PRAME-Specific**	
NCT02743611	n.d.
NCT03503968/EudraCT-2017-000440-18	n.d.
EudraCT-2018-000717-20	n.d.
**MiHA HA-1H-Specific**	
EudraCT-2010-024625-20 NTR3454/NL3307	HA-1H TCR-transduced CMV or EBV-specific T-cell products could not be generated for 4 out of 9 patients; TCR-T cells could not be detected (lack of TCR-T-cell expansion) in peripheral blood in 2 out of 5 treated patients at any time during follow-up; 3 out of 5 treated patients died during follow-up for causes not related to treatment; overall feasibility and efficacy of the procedure was too low to warrant further developments of this therapy
NCT04464889/EudraCT-2019-002346-20	n.d.
NCT03326921	n.d.
**Other**	
NTR6541/NL6357	n.d.

Abbreviations: AML, acute myeloid leukemia; CMV, cytomegalovirus; EBV, Epstein–Barr virus; HA-1H, HLA-A*02:01-restricted minor histocompatibility antigen 1 peptide variant H; MiHA, minor histocompatibility antigen; n.d., no data; PRAME, preferentially expressed antigen in melanoma; TCR, T-cell receptor; WT1, Wilms’ tumor 1. Last search on 25 July 2021.

Multiple antigens for AML have been described over the years that have yet to be explored in the clinical setting as target antigens in TCR-T-cell therapy [7,8]. For some of these antigens, preclinical and clinical data hinted at their potential role as targets for TCR-T-cell therapy, including proteinase 3 (PR3), hyaluronan-mediated motility receptor (HMMR), and T-cell receptor γ chain alternate reading frame protein (TARP). Together with WT1 and PRAME, PR3 is an AML-associated antigen overexpressed in AML blasts [36,84]. These antigens are differentially expressed in leukemic stem cells (LSCs) compared to hematopoietic stem cells; however, PR3 diverged from the other antigens analyzed in that it was comparatively more expressed on bulk leukemic cells rather than LSC [85]. Cytotoxic T-cell responses against PR3 are spontaneously detected in AML patients [40] and after PR1 vaccination [86,87]. PR1 is a nonameric HLA-A*02:01-restricted peptide derived from PR3 and neutrophil elastase (NE) that is commonly found to be overexpressed in AML [88]. PR3-specific T-cell activity has also been observed after allo-HSCT [89,90,91], although in this case, LAA-specific T cells are difficult to detect in early phases after transplantation [92]. Results from clinical trials using allo-HSCT demonstrated that donor T cells mediate PR3-directed anti-AML responses, including in treated R/R AML patients [89,90]. Nonetheless, cytotoxic T-cell responses against PR3 were characterized by low-affinity interactions against this self-antigen [89]. Moreover, administration of PR1-specific bulk T cells into an AML xenograft NOD/SCID mouse model led to the reduction of human AML cells in mice [93]. Despite the aforementioned encouraging data, to the best of our knowledge, no TCRs have been isolated from PR1-specific T-cell clones and used in TCR-T-cell therapies. Current strategies targeting PR3 in the context of HLA-A2 restriction are mostly based on anti-PR1/HLA-A2 antibodies [94,95], including bi-specific antibodies [96] and TCR-like CARs [97]. This warrants the potential of this antigen in fighting AML using TCR engineered T cells.

Greiner and colleagues defined HMMR, also known as RHAMM or CD168, as an immunogenic AML-associated antigen that could be targeted in immunotherapies [98]. Initially considered a promising antigen expressed in blasts in a considerable number of AML patients, HMMR expression was associated with poor overall survival and, therefore, also considered a potential prognostic factor [99]. DCs transfected with *HMMR*-encoding mRNA elicited HMMR-specific TCR responses [100]. However, the upregulation of HMMR via mRNA transfection showed no additional benefit compared to unmodified cells, as DCs presented basal HMMR expression levels to sufficiently activate T cells. T-cell reactivities against HMMR’s R3 peptide have been detected in AML patients after standard of care [101,102,103], including after HMMR-R3 peptide vaccination [104,105]. However, in some cases, HMMR-specific T cells were non-functional [103]. T cells modified to express HMMR-specific TCRs were able to recognize AML target cells in a humanized xenograft mouse model leading to reduced tumor burden [106]. The addition of IL-15 further enhanced the antitumor effect of TCR-T cells. However, HMMR-specific TCR-T cells also recognized hematopoietic stem cells (HSCs), which restricted the use of these cells to MHC-mismatched HSC transplantation. In addition, Snauwaert and colleagues pointed out that HMMR may not be suitable as a candidate antigen in AML due to similar expression levels of HMMR in LSCs and hematopoietic stem cells in healthy individuals and its upregulation in activated T cells [107]. Therefore, the further development of HMMR-specific TCR-T-cell therapies seems unlikely. With regards to TARP, this antigen is upregulated in AML cell lines and de novo pediatric and adult AML cells [108]. Moreover, overexpression of an alternative TARP transcript is specific to AML, being absent in other types of leukemia, such as B-ALL and CML cells [108]. More importantly, TARP-specific TCR-T cells exert cytotoxic activity against TARP-positive AML cell lines and primary cells [108]. Other reports suggest other candidates for TCR-T-cell immunotherapy in AML, such as myeloperoxidase (MPO) [109] or human telomerase reverse transcriptase (hTERT) [110]. In these two cases, high-avidity TCRs were used.

Due to difficulties in isolating high-affinity/high-avidity TCRs for self-antigens, such as those mentioned above, novel target antigens are required. Donor T cells in HSCT can mediate both GVHD and GVL effects. The capacity of these T cells to recognize mismatched HLA molecules makes them a valuable tool in TCR-T therapy for relapsed patients after allo-HSCT. Similar to HA-1 antigen, HLA-DPB1-reactive T cells have been described as potent GVL inducers [111,112]. To prevent recurrent disease after allogeneic HSCT, T cells engineered with TCRs against HLA-DPB1 antigens, which are mismatched in many allogeneic transplantations, recognized AML cells in vitro; however, only TCR-engineered CD4 T cells were able to effectively mediate leukemic elimination in vivo [113]. Extensive screening and validation of TCRs seem critical in this context to avoid targeting HLA-DP antigens that are also expressed on healthy tissues that could lead to undesirable GVHD [113]. In some cases, due to genetic aberrations that are characteristic of AML, new point mutations may occur in specific genes leading to the formation of neoantigens that the immune system can target more efficiently than self-antigens. An example of a neoantigen in AML is the CBFB-MYH11 fusion protein. A recent report showed that HLA-B*40:01-restricted T-cell clones react with high avidity against a nonameric peptide from the CBFB-MYH11 fusion protein [114]. These T cells showed antitumor reactivity against AML cell lines and primary AML cells both in vitro and in vivo in a humanized xenograft mouse model. Moreover, the introduction of TCRs derived from CBFB-MYH11-specific T-cell clones showed anti-leukemic activity in vitro. T-cell responses have also been observed against nucleophosmin 1 (*NPM1*) mutations, which occur in approximately 30% of AML patients [115,116,117]. Isolation and transduction of an HLA-A*02:01-restricted mutated NPM1 peptide-specific TCR into T cells resulted in anti-AML activity in vivo [117]. Finally, the expression of inhibitory immune checkpoints in T cells is a factor to consider in TCR-T-cell therapy. The increased expression of immune checkpoints, such as programmed death 1 (PD-1), cytotoxic T-lymphocyte antigen 4 (CTLA4), T-cell immunoglobulin and mucin-domain containing-3 (TIM-3), and lymphocyte-activation gene 3 (LAG3), contribute to AML immune evasion and are associated with disease progression and relapse (reviewed by [118]). Thus, disruption of inhibitory receptors expressed by TCR-T cells and/or combinatorial immunotherapies based on TCR-T cells and immune checkpoint inhibitors (as seen in the combination of chemotherapy or hypomethylating agents) may pave the way for a higher efficacy and reduced immune evasion in the context of TCR-T-cell therapy for AML [119].

## 6. Conclusions

In summary, results from current clinical trials using TCR-T-cell therapy for AML indicate no treatment-related toxicities. This is achieved thanks to the careful selection of high-affinity TCRs derived from natural repertoires that do not rely on artificially enhancing TCR affinity above physiological levels. In terms of efficacy, it is too soon to draw conclusions from these studies, given their early phase design and the results from most of them being pending. However, preliminary results indicate that this type of therapy may be efficacious for AML, especially in cases of relapse, where remaining leukemic blasts may not be eliminated with the standard of care, warranting the development of improved TCR-T-cell strategies to further increase their clinical benefit.
